# Circulating ANGPTL3 and ANGPTL4 levels predict coronary artery atherosclerosis severity

**DOI:** 10.1186/s12944-021-01580-z

**Published:** 2021-11-06

**Authors:** Ting Sun, Wanlin Zhan, Lijiang Wei, Zuojun Xu, Li Fan, Yang Zhuo, Changqian Wang, Junfeng Zhang

**Affiliations:** Department of Cardiology, Shanghai Ninth People’s Hospital, Shanghai Jiao Tong University School of Medicine, Shanghai, 200025 China

**Keywords:** ANGPTL3, ANGPTL4, Coronary atherosclerosis, Predictors

## Abstract

**Background:**

We investigated the role of ANGPTL3 and ANGPTL4 in atherosclerosis development and determined whether plasma concentrations of ANGPTL3 and ANGPTL4 are related to the degree of coronary stenosis.

**Methods:**

A total of 305 consecutive patients with angina who underwent diagnostic coronary angiography were enrolled in the study between August 2017 and August 2018. The levels of ANGPTL3 and ANGPTL4 were measured by using competitive ELISA kits.

**Results:**

According to the degree of coronary artery stenosis, patients were classified into four types: coronary artery stenosis of < 10%, 10-50%, 50-75, and > 75%. The plasma ANGPTL3 level was higher (51.71 ± 52.67 vs. 24.65 ± 10.32 ng/mL, *P* < 0.001) and that of ANGPTL4 was lower (454.66 ± 269.05 vs. 875.49 ± 961.15 ng/mL, *P* < 0.001) in the coronary artery stenosis ≥ 10% group than in the < 10% group. ANGPTL3 and ANGPTL4 levels were significantly associated with the severity of coronary vascular stenosis. ROC curve analyses indicated that ANGPTL3 concentrations above 30.5 ng/mL can predict atherosclerosis with a sensitivity of 71.2% and specificity of 75.3%, and that ANGPTL4 levels below 497.5 ng/mL can predict atherosclerosis with a sensitivity of 63.9% and specificity of 74.5%. ANGPTL3 and ANGPTL4 were determined to be independent risk factors for coronary atherosclerosis with odds ratios (ORs) of 0.189 (95% CI 0.097-0.368, *P* < 0.001) and 3.625 (95% CI 1.873-7.016, *P* < 0.001), respectively.

**Conclusions:**

Increased ANGPTL3 or decreased ANGPTL4 shows an association with coronary atherosclerosis and, may become a predictor of coronary atherosclerosis in the future.

**Supplementary Information:**

The online version contains supplementary material available at 10.1186/s12944-021-01580-z.

## Introduction

Coronary heart disease (CHD), also called coronary artery disease and atherosclerotic heart disease, is the end result of the accumulation of atheromatous plaques within the walls of the coronary arteries, causing myocardial ischemia, hypoxia and even necrosis [1]. The incidence of CHD is still rising, and CHD is the leading global cause of mortality. The death rate of CHD has decreased because effective therapeutic approaches have been developed, such as the application of intravascular imaging, the use of drug-eluting stents [2], P2Y_12_ inhibitor monotherapy [3], prolonged dual antiplatelet therapy [4] and so on.

Dyslipidemia, such as increased low-density lipoprotein-cholesterol (LDL-C) and decreased high-density lipoprotein-cholesterol (HDL-C), is related to the development of atherosclerosis [5–10]. Increased LDL-C is commonly useful in identifying high-risk CHD, but some patients with coronary atherosclerosis may have normal serum lipid levels. We infer that there may be dysfunction of lipid regulation before changes in serum lipids and serum lipoprotein become apparent. It is of necessary clinical significance to identify new indicators for the early identification of the high-risk coronary atherosclerosis population having normal serum lipid levels.

Some researchers have worked out several diverse mechanisms of dyslipidemia. Angiopoietin-like proteins (ANGPTLs) were found to be structurally and functionally similar to the angiopoietins in 1998 at first, and until 2005, they were noticed to play a role in the regulation of lipids [11]. To date, eight ANGPTLs have been discovered, of which ANGPTL3 and ANGPTL4 are the most frequently mentioned in lipid metabolism. ANGPTL3 is an endogenous inhibitor of lipoprotein lipase (LPL) and endothelial lipase (EL) [11, 12]. The role of ANGPTL3 in regulating LPL and EL activity has been well established. LPL, characterized as a triglyceride hydrolase, primarily hydrolyzes triglycerides in the neutral lipid core of chylomicrons and very low-density lipoprotein (VLDL), thereby releasing unesterified fatty acids [11]. In 2007, EL was reported to have phospholipase A1 activity and regulate the level of HDL-C [13]. ANGPTL3 activity regulates triglycerides and HDL-C clearance partly by disinhibiting LPL and EL [11–13]. In the last ten years, it has been proven that inactivation or variants of the ANGPTL3 gene in humans and mice induce a marked reduction in the levels of plasma triglycerides and cholesterol-carrying lipoproteins, including VLDL-C, LDL-C and HDL-C [14–17]. Based on a phase 3 randomized clinical trial with an estimated enrollment of 10,000 participants, hypertriglyceridemia (HTG) is causally linked to CHD and reducing HTG can significantly reduce cardiovascular events in patients with diabetes [18]. ANGPTL3-targeted therapies to reduce triglycerides are under development. Recently, anti-ANGPTL3 therapies have been shown to effectively lower lipids levels based on the results of phase I clinical trials with a monoclonal anti-ANGPTL3 antibody and anti-sense oligonucleotide (ASO) [16, 17, 19].

ANGPTL4 is also an inhibitor of LPL [11, 20–22]. When ANGPTL4 is overexpressed, the activity of LPL is suppressed; as a result, triglycerides clearance is decreased, leading to an increase in triglycerides [23]. However, some studies have revealed a reduction in plasma triglyceride levels in mice with ANGPTL4 deficiency and inactivation [24, 25]. In accordance with the findings of ANGPTL4-mediated regulation of lipid metabolism in mice, carriers of loss-of-function mutations in ANGPTL4 have lower triglyceride levels and higher HDL-C levels than noncarriers [26–28]. In 2013, Georgiadi A et al. reported that ANGPTL4 retards atherosclerotic plaque progression by reducing the inflammatory response to saturated fats, which is independent of its effect on plasma lipid levels [29].

As mentioned above, the roles of ANGPTL4 in CHD present conflicting results in different studies. The roles of ANGPTL3 are mostly focused on acute myocardial infarction (AMI), rarely in atherosclerosis. The role of ANGPTL4 in atherosclerosis development is unclear. Does ANGPTL3 or ANGPTL4 represent a better predictive indicator for coronary atherosclerosis? To answer this question, we performed a study of ANGPTL3 and ANGPTL4 in a cohort of patients with angina to clarify the associations of these proteins with coronary atherosclerosis severity.

## Materials and methods

### Study population and data collection

A total of 305 consecutive patients with angina, referred to the Department of Cardiology or Emergency Medicine of Shanghai Ninth People’s Hospital between August 2017 and August 2018, were enrolled in this study. The inclusion and exclusion criteria of participants are shown in Table 1. All data were obtained from electronic medical records or charts in the hospital, and included age, sex, smoking, family history and so on. Ethical approval of this study protocol was given by the Shanghai Ninth People’s Hospital’s ethics committee and research board. Prior to study participation, written informed consent was signed by all study participants or the families of the patients. The study was part of a project for construction and application of a biobank for coronary heart disease at Shanghai Ninth People’s Hospital (YBKA201910).

**Table 1:** Inclusion and exclusion criteria of participants

Inclusion criteria	Exclusion criteria
1) over 18 years old; 2) angina; 3) silent myocardial ischemia; 4) all patients undergoing invasive coronary angiography.	1) acute myocardial infarction; 2) severe hepatic or renal dysfunction; 3) pulmonary heart diseases; 4) valvular heart diseases; 5) infective endocarditis; 6) malignant tumors; 7) hyperthyroidism.

### Blood collection, biochemical and anthropometric measurements

Blood samples were pre-heparin, taken after a 12-h overnight fast on the day before coronary angiography, and collected in vacutainer EDTA tubes. After 30 min of clotting at room temperature, the samples were then centrifuged at 3000 rpm for 20 min at 4 °C. The supernatant liquids were collected in small aliquots and stored in a − 80 °C deep freezer until assayed. The serum lipids were enzymatically measured on a Hitachi 747 chemical analyzer (Hitachi, Tokyo, Japan). Fasting blood glucose (FBG), myoglobin, brain natriuretic peptide (BNP) and cTnI were measured using standard laboratory techniques. Transthoracic echocardiography (TTE) was performed in all patients by using an ultrasound device (ie33, Philips Medical System, Bothell, Washington, USA). The left ventricular ejection fraction (LVEF) was measured and calculated.

### Determinations of ANGPTL3 and ANGPTL4 concentrations

Plasma ANGPTL3 and ANGPTL4 levels were determined using enzyme-linked immunosorbent assay (ELISA) kits (R&D Systems, Minnelis, MN, USA) according to the manufacturer’s instructions. The plasma samples were diluted 1:4 in sample diluent for detection. The samples or standards and biotin antigen were added at 50 μL/well to a 96 well plate. Then the plate was incubated for 30 min at 37 °C. After washing five times with 300 μL/well PBST (50 mM potassium phosphate 150 mM NaCl, 0.15% Tween 20; pH 7.4), avidin-HRP was added at 50 μL/well (100 μL avidin-HRP solution diluted to 6 mL) and the plate was incubated for another 30 min at 37 °C. Following five washes, 50 μL/well coloration liquids A and B were added, and the plate was incubated in the dark for 10 min at 37 °C. The plates were analyzed within 10 min using an ultraviolet spectrophotometer (Multiskan GO, Thermo Scientific) at 450 nm. The standard curve was constructed by plotting the concentration (X) of standards against the mean absorbance (Y) of standards at 450 nm (Supplementary Fig. 1). A logistic equation was used to fit the standards and calculate the sample concentration.

### Diagnostic coronary angiographic examinations and groups

Coronary angiography was performed by two expert cardiologists blinded to the blood test results. Of the 66 participants with borderline coronary artery lesions, 10 underwent intravenous ultrasound (IVUS) imaging, and 7 underwent OCT. Based on the international statistical classification of disease and related health problems (10th Revision) and the American Heart Association classification of cardiovascular diseases, patients were screened and classified into four types: 1) nonstenotic coronary arteries, with coronary stenosis < 10% in diameter; 2) coronary atherosclerosis, with one or more coronary stenoses 10 - 50% in diameter; 3) CHD, one or more coronary stenoses 50 - 75% in diameter; and 4) CHD, one or more coronary stenoses ≥ 75% in diameter in any three coronary arteries (left anterior descending (LAD), left circumflex (LCX), right coronary artery (RCA)).

### Statistical analysis

The statistical analysis was performed using IBM SPSS version 22 statistical package for Windows. A two-sided *P*-value of < 0 .05 was considered statistically significant. Continuous variables are expressed as the mean ± SD or medians with interquartile ranges, and categorical variables are expressed as frequencies (percentages). A Student’s t-test or one-way ANOVA was used to evaluate continuous variables with normal distribution, and the Mann-Whitney U-test was used to evaluate continuous variables with skewness distribution. The chi-square test was used to evaluate categorial variables. Spearman correlation was performed to estimate the relationships between the plasma ANGPTL3 or ANGPTL4 levels and other biochemical parameters. A receiver operating characteristic curve for ANGPTL3 or ANGPTL4 was plotted to illustrate the diagnostic power of ANGPTL3 or ANGPTL4 for coronary stenosis patients. The optimal cut-offs were obtained by measuring the size of the area under the ROC curve (AUC). Univariate and multivariate logistic regression models were performed to distinguish the risk factors for CHD.

## Results

### Subject characteristics

A total of 305 patients (157 males, 148 females) were recruited, for whom the demographic, biochemical and clinical characteristics are shown in Table 2. The plasma ANGPTL3 level was higher and that of ANGPTL4 lower in the coronary stenosis > 10% group than in the nonstenosis group. Compared to the nonstenosis group, the coronary stenosis over 10% group had more elderly patients; males; overweight patients; smokers; patients with diabetes mellitus, hypertension or New York Heart Association (NYHA) class III or IV (2013 ACC/AHA guidelines); and patients using hypoglycemic drugs and antihypertensive drugs. There was no significant difference in alcohol consumption, lipid levels, CHD history, atrial fibrillation, hypolipidemic drugs or aspirin use between the two groups.

**Table 2:** Demographic, clinical and biochemical characteristics of the study sample according to the results of coronary arteriography

Variable	The stenosis of coronary artery		χ, *t, Z* value	*P* value
	≥ 10% (*n* = 208)	< 10% (*n* = 97)		
Age, years	67.52 ± 9.91	61.86 ± 9.59	3.615	< 0.001
Sex (male), %	55.8 (116/208)	42.3 (41/97)	4.827	0.028
Smokers, %	39.4 (82/208)	25.8 (25/97)	5.412	0.020
Alcohol consumers, %	22.6 (47/208)	18.6 (18/97)	0.644	0.422
BMI ≥ 24 kg/m^2^, %	63.2 (129/204)	50.5 (48/95)	4.334	0.043
CHD family history, %	61.5 (128/208)	53.6 (52/97)	2.191	0.212
Diabetes mellitus, %	26.4 (55/208)	11.3 (11/97)	8.897	0.003
Hypertension, %	65.9 (137/208)	48.5 (47/97)	8.379	0.004
NYHA class III/ IV, %	10.1 (21/208)	3.1 (3/97)	6.491	0.011
Atrial fibrillation, %	9.1 (19/208)	7.2 (7/97)	0.312	0.576
Hypolipidemic drugs, %	18.2 (37/203)	25.0 (24/96)	1.841	0.175
Aspirin use, %	91.8 (191/208)	93.8 (91/97)	0.375	0.540
Hypoglycemic drugs, %	22.1 (46/208)	9.3 (9/97)	7.375	0.007
Antihypertensive drugs, %	59.6 (124/208)	41.2 (40/97)	8.988	0.003
Blood glucose, mmol/L	6.04 ± 2.08	5.78 ± 2.28	0.006	0.370
GHb, %	6.43 ± 1.13	6.98 ± 6.92	4.976	0.350
Myoglobin, μg/L	44.71 ± 49.66	31.41 ± 22.94	9.192	0.002
cTnI, ng/mL	0.13 ± 1.01	0.03 ± 0.09	−0.349	0.727
LVEF, %	61.32 ± 6.98	61.08 ± 6.24	0.410	0.800
LVEDP, mmHg	6.16 ± 2.31	6.63 ± 2.06	2.202	0.140
Heart Rate, bpm	75.39 ± 16.17	76.98 ± 17.05	1.317	0.433
BNP, *p*g/mL	155.09 ± 354.50	171.21 ± 516.34	−1.695	0.090
Lipoprotein(a), g/L	0.16 ± 0.16	0.16 ± 0.19	3.162	0.728
apoE, mg/dL	4.52 ± 1.91	4.52 ± 1.54	0.382	0.997
FFA, mmol/L	0.51 ± 0.21	0.47 ± 0.20	1.226	0.214
LDL-C, mmol/L	2.74 ± 0.91	2.92 ± 0.82	0.631	0.118
HDL-C, mmol/L	1.07 ± 0.31	1.11 ± 0.33	0.000	0.303
Triglycerides, mmol/L	1.68 ± 0.99	1.60 ± 0.97	0.700	0.543
apoA-I, g/L	1.12 ± 0.20	1.14 ± 0.22	0.050	0.447
apoB, g/L	0.85 ± 0.25	0.94 ± 0.73	0.842	0.175
apoA-I/apoB	1.48 ± 0.52	1.45 ± 0.41	0.777	0.709
ANGPTL3, ng/mL	51.71 ± 52.67	24.65 ± 10.32	−8.027	< 0.001
ANGPTL4, ng/mL	454.66 ± 269.05	875.49 ± 961.15	−4.683	< 0.001

*CHD* coronary heart diseases, *NYHA* New York Heart Association (2013 ACC/AHA guidelines), *GHb* glycosylated hemoglobin, *LVEF* left ventricular ejection fraction, *LVEDP* left ventricular end diastolic pressure, *BNP* B-type natriuretic peptide, *apo* apolipoprotein, *FFA* free fatty acid, *LDL-C* low-density lipoprotein-cholesterol, *HDL-C* high-density lipoprotein-cholesterol, *BMI* body mass index, *ANGPTL* angiopoietin-like proteins;

### ANGPTL3 and ANGPTL4 levels and coronary stenosis severity

The levels of ANGPTL3 and ANGPTL4, and blood lipids in the different stenosis groups are shown in Table 3. Compared to that in the nonstenosis group, the concentration of ANGPTL3 significantly increased in all of the coronary stenosis groups (*P* < 0.01). However, the ANGPTL3 levels did not different among the various stenosis groups. ANGPTL4 levels were obviously decreased in the anycoronary stenosis groups compared with the nonstenosis group. The blood lipid index among these various stenosis groups, was not different except for free fatty acids (FFAs). The concentrations of ANGPTL3 or ANGPTL4 and lipid levels in the different groups varied according to the number of involved vessels with stenosis ≥ 50% (Table 4). The ANGPTL3 level was significantly increased (*P* < 0.01) and the ANGPTL4 level was decreased (*P* < 0.05) in patients with multivessel lesions compared with single vessel-involved patients. The level of ANGPTL3 or ANGPTL4 in the different coronary stenosis severity based on sex are shown in Supplementary Table 1, and no statistically significant differences are found between the sexes.

**Table 3:** ANGPTL3, ANGPTL4 and lipids levels of the study sample according to the degree of coronary artery stenosis

Variable	The degree of coronary artery stenosis							
	< 10%	10-50%	50-75%	≥ 75%	*F* or *Z* value	*P* value	*t* or *Z* value	*P*_*1*_ value
ANGPTL3, ng/mL	24.66 ± 10.32	51.90 ± 68.68	51.56 ± 31.59	51.56 ± 38.81	67.073	< 0.001	−6.106	< 0.001
ANGPTL4, ng/mL	875.5 ± 961.2	465.4 ± 268.6	472.6 ± 309.1	409.0 ± 202.8	22.733	< 0.001	−3.731	< 0.001
Triglycerides, mmol/L	1.60 ± 0.97	1.67 ± 0.94	1.72 ± 1.10	1.65 ± 0.92	0.176	0.913	1.005	0.658
LDL-C, mmol/L	2.92 ± 0.81	2.72 ± 0.86	2.69 ± 0.99	2.86 ± 0.90	1.179	0.318	0.207	0.119
HDL-C, mmol/L	1.11 ± 0.33	1.12 ± 0.34	1.01 ± 0.26	1.03 ± 0.32	1.938	0.124	0.110	0.798
apoA-I, g/L	1.14 ± 0.22	1.14 ± 0.18	1.10 ± 0.19	1.09 ± 0.25	0.721	0.540	0.297	0.930
apoB, g/L	0.94 ± 0.73	0.86 ± 0.25	0.83 ± 0.26	0.85 ± 0.24	0.661	0.577	0.400	0.441
apoA-I/apoB	1.45 ± 0.41	1.49 ± 0.48	1.52 ± 0.58	1.42 ± 0.48	0.323	0.809	0.135	0.667
apoE, mg/dL	4.52 ± 1.54	4.45 ± 1.24	4.73 ± 2.77	4.36 ± 1.29	0.333	0.801	0.462	0.776
Lipoprotein(a), g/L	0.16 ± 0.19	0.15 ± 0.15	0.15 ± 0.16	0.19 ± 0.19	0.554	0.646	4.016	0.451
FFA, mmol/L	0.47 ± 0.20	0.47 ± 0.17	0.47 ± 0.21	0.63 ± 0.23	6.429	< 0.001	0.006	0.964

*P*, comparison among the four groups; *P*_*1*_, comparison between non-stenosis group and stenosis 10 - 50% group; *ANGPTL* angiopoietin-like proteins, *LDL-C* low-density lipoprotein-cholesterol, *HDL-C* high-density lipoprotein-cholesterol, *apo* apolipoprotein, *FFA* free fatty acid; ANGPTL3,4 values for Mann-Whitney U-test; lipid variables values for one-way ANOVA and A Student’s t-test

**Table 4:** ANGPTL3, ANGPTL4 and lipids levels of the study sample according to the number of involved vessels stenosis ≥ 50%

Variable	The number of involved vessels stenosis (≥ 50%)				
	0-vessel	1-vessel	≥ 2-vessel	*F* value	*P* value
ANGPTL3, ng/mL	38.10 ± 50.54	40.21 ± 22.33	57.37 ± 38.24	37.708	< 0.001
ANGPTL4, ng/mL	669.73 ± 738.11	456.09 ± 286.90	445.31 ± 251.73	7.480	0.024
Triglycerides, mmol/L	1.65 ± 0.96	1.77 ± 1.18	1.47 ± 0.57	1.094	0.336
LDL-C, mmol/L	2.81 ± 0.84	2.69 ± 0.92	2.92 ± 1.01	0.905	0.406
HDL-C, mmol/L	1.11 ± 0.33	1.03 ± 0.28	1.02 ± 0.29	2.469	0.087
apoA-I, g/L	1.14 ± 0.20	1.10 ± 0.20	1.07 ± 0.24	1.624	0.200
apoB, g/L	0.91 ± 0.57	0.82 ± 0.24	0.85 ± 0.28	0.655	0.520
apoA-I/apoB	1.47 ± 0.44	1.50 ± 0.52	1.44 ± 0.59	0.106	0.900
apoE, mg/dL	4.51 ± 1.40	4.74 ± 2.62	4.15 ± 1.38	0.958	0.385
Lipoprotein(a), g/L	0.16 ± 0.18	0.14 ± 0.15	0.20 ± 0.20	1.844	0.160
FFA, mmol/L	0.47 ± 0.19	0.53 ± 0.23	0.53 ± 0.24	1.540	0.217

Values are expressed as mean ± standard deviation or percentage; *ANGPTL* angiopoietin-like proteins, *LDL-C* low-density lipoprotein-cholesterol, *HDL-C* high-density lipoprotein-cholesterol, *apo* apolipoprotein, *FFA* Free fatty acid

### Relationships between ANGPTL 3, ANGPTL4 and clinically associated factors

**Fig. 1:**
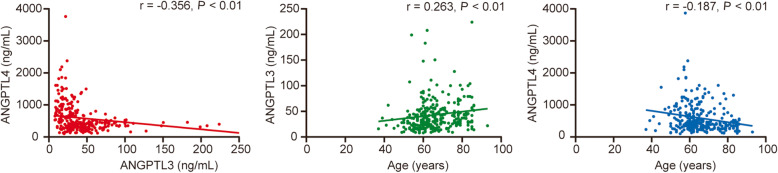
Association between serum ANGPTL3 or ANGPTL4 levels and age

The relationships between ANGPTL3 or ANGPTL4 levels and CHD factors are shown in Fig. 1, Supplementary Table 2 and Supplementary Table 3.There was no significant difference between the plasma levels of ANGPTL3 or ANGPTL4 and lipid parameters. As shown in Fig. 1, the concentration of ANGPTL3 was negatively associated with that of ANGPTL4 and positively associated with age, but ANGPTL4 had a negative association with age. There were lower levels of ANGPTL4 in the hypertension group (541.78 ± 453.35 vs. 659.54 ± 799.80 ng/mL, *P* < 0.05) and the old-aged group (541.10 ± 717.23 vs. 632.02 ± 504.20 ng/mL, *P* < 0.05) than in their control groups (Supplementary Table 2).

### ROC curve analysis for ANGPTL3, 4 levels

**Fig. 2:**
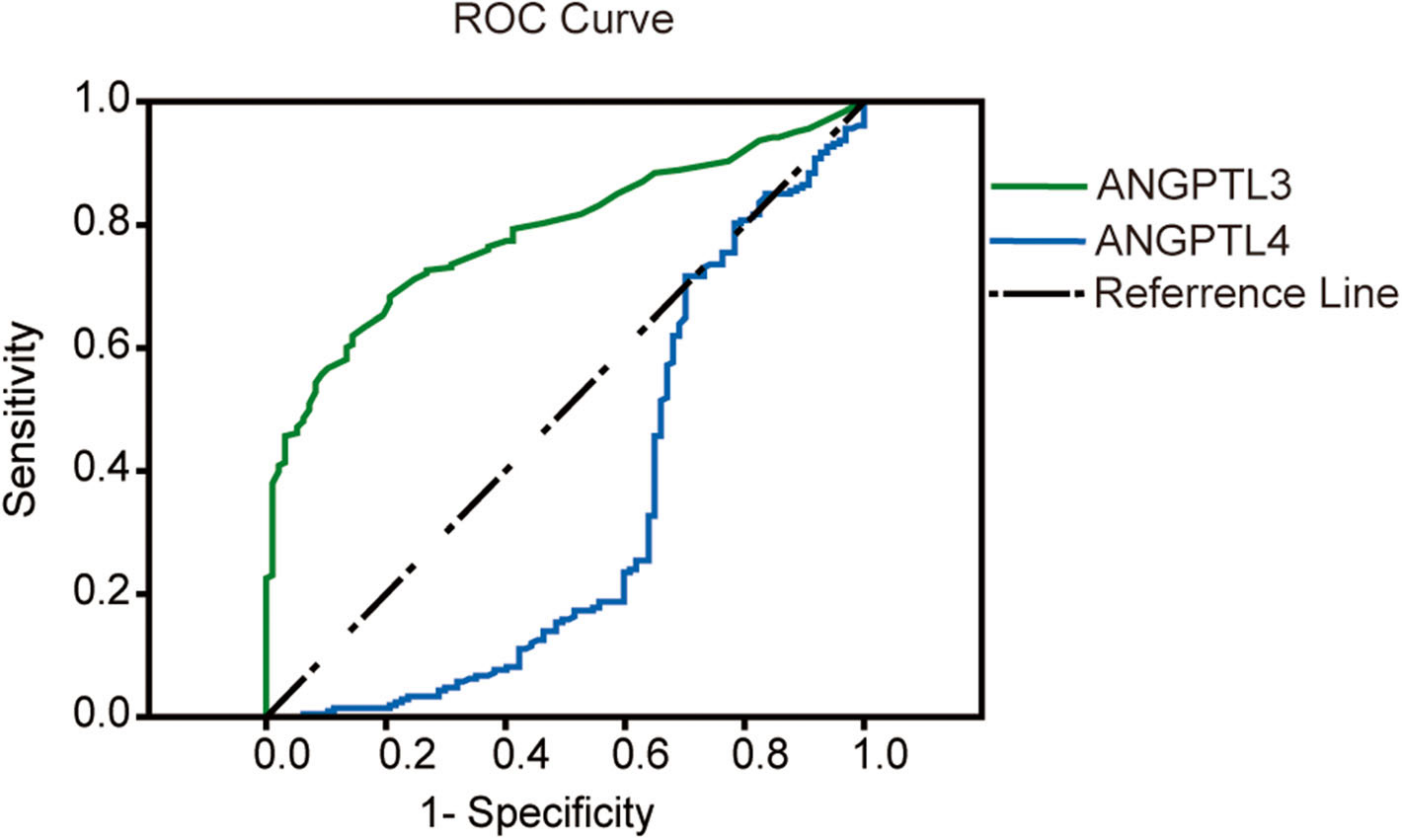
Receiver-operating characteristic (ROC) curves for ANGPTL3 and ANGPTL4 in predicting atherosclerosis

Figure 2 and Supplementary Table 4 show the cut-off concentrations for ANGPTL3 and ANGPTL4, which differentiate between coronary atherosclerosis and nonstenotic coronary arteries, respectively. Using 30.5 ng/mL as a cutoff value, the sensitivity and specificity of ANGPTL3 for coronary atherosclerosis were 71.2 and 75.3%, and the area under the ROC curve was 0.785 (*P* < 0.01). The optimal ANGPTL4 cutoff point for coronary atherosclerosis was 497.5 ng/mL with a sensitivity of 63.9% and specificity of 74.5%. In addition, the participants were divided into two groups based on the cutoff point of ANGPTL3 or ANGPTL4, and CHD factors were analyzed, shown in Supplementary Table 5. More elderly individuals (56% vs. 38%, *P* < 0.01) and more individuals with (Lipoprotein(a) < 0.3 g/L) (91% vs. 75%, *P* < 0.01) were seen in the ANGPTL3 > 30.5 ng/mL group than that in the ANGPTL3 < 30.5 ng/mL group. There were more elderly individuals (55% vs. 36%, *P* < 0.01) and more patients with hypertension (65% vs. 53%, *P* < 0.05) in the ANGPTL4 < 497.5 ng/mL group than that in the ANGPTL4 > 497.5 ng/mL group.

### Predictive value of ANGPTL3, and ANGPTL4 for atherosclerosis

Univariate and multivariate regression analyses were performed to identify the risk factors for coronary atherosclerosis, and the results are shown in Table 5. Increasing age, sex, higher body mass index, smoking, diabetes, hypertension, elevated ANGPTL3 and decreased ANGPTL4 had a statistically significant association with atherosclerosis development in the univariable analysis. However, in the multivariable logistic regression analysis, ANGPTL3 and ANGPTL4 were determined to be independent risk factors for coronary atherosclerosis with odds ratios (ORs) of 0.189 (95% CI 0.097-0.368, *P* < 0.001) and 3.625 (95% CI 1.873-7.016, *P* < 0.001) respectively. Smoking and diabetes mellitus (DM) are independent risk factors for CHD. The clinical characteristics and CHD factors according to smoking status and DM are shown in Supplementary Table 6. There were more elderly people, hypertension, more heart failure and lower HDL-C levels in the DM group, and more male patients, more alcohol consumers, fewer elderly people, and lower HDL-C and LVEF levels in the smoker group. However, there was no difference in ANGPTL3 or ANGPTL4 between the smoker and nonsmoker groups or between the diabetes and nondiabetes groups.

**Table 5:** Univariate and multivariate logistic regression model for prediction of coronary atherosclerosis

Variable	Univariate analysis OR (95% CI)	*P* value	Multivariate analysis OR (95% CI)	*P* value
Age	0.940 (0.915-0.967)	< 0.001	0.591 (0.303-1.153)	0.123
Smoke	1.874 (1.100-3.195)	0.021	3.120 (1.440-6.757)	0.004
Diabetes mellitus	0.356 (0.177-0.716)	0.004	0.429 (0.181-1.014)	0.054
Hypertension	2.053 (1.257-3.353)	0.004	1.803 (0.916-3.551)	0.088
Overweight	0.732 (0.446-1.202)	0.217	0.926 (0.477-1.796)	0.819
CHD family history	0.708 (0.435-1.155)	0.167	1.767 (0.924-3.377)	0.085
LDL-C	1.559 (0.928-2.620)	0.093	1.198 (0.617-2.328)	0.594
HDL-C	1.472 (0.881-2.459)	0.140	1.109 (0.550-2.237)	0.773
ANGPTL3	0.138 (0.080-0.238)	< 0.001	0.189 (0.097-0.368)	< 0.001
ANGPTL4	5.181 (3.084-8.702)	< 0.001	3.625 (1.873-7.016)	< 0.001

Coronary atherosclerosis: with one or more coronary stenosis 10 - 50% in diameter; *BMI* body mass index, *Overweight* BMI ≥ 24 kg/m^2^, *CHD* coronary heart diseases, *LDL-C* low-density lipoprotein-cholesterol, *HDL-C* high-density lipoprotein-cholesterol, *ANGPTL* angiopoietin-like proteins

## Discussion

Given the regulation of ANGPTL3 and ANGPLT4 in lipid metabolism, there is growing interest regarding their correlations with CHD. At present, it is recognized that inhibition of LPL by ANGTL3 may increase the plasma levels of LDL-C and VLDL-C. Meanwhile, high serum levels of LDL-C and VLDL-C may induce the formation of foam cells and the proliferation and migration of vascular smooth muscle cells, which lead to atherosclerosis [14–17]. However, the specific mechanism of action of ANGPTL4 in the occurrence and development of atherosclerosis is not yet distinct. Previous studies have shown that ANGPTL4 is related to vascular endothelial integrity [30], the inflammatory response [29], oxidative stress [29], neovascularization [31] and other proatherosclerosis factors. As the study showed, the concentration of ANGPTL3 was significantly higher and that of ANGPTL4 was obviously lower in the atherosclerosis group than in the nonstenosis group. However, lipid parameters were not significantly different between the two groups. These results suggested that ANGPTL3 or ANGPTL4 is superior to lipid parameters alone in evaluation of coronary atherosclerosis.

Both ANGPTL3 and ANGPTL4 were significantly related to coronary atherosclerosis. A concentration of ANGPTL3 over 30.5 ng/mL had a better sensitivity and specificity for the prediction of coronary atherosclerosis (sensitivity = 71.2%, specificity = 75.3%). On the contrary, a plasma concentration of ANGPTL4 below 497.5 ng/mL could be used to predict the occurrence of coronary atherosclerosis (sensitivity = 63.9%, specificity = 74.5%). Increased ANGPTL3 and decreased ANGPTL4 were positively associated with the progression of atherosclerosis, and ANGPTL3 had negative associations with ANGPTL4. All these data revealed that ANGPTL3 and ANGPTL4 had the opposite effect on atherosclerotic development. Moreover, ANGPTL3 and ANGPTL4 may act as independent predictors of coronary atherosclerosis.

Age was also an independent risk factor for coronary atherosclerosis. Of all the risk factors we studied (Supplementary Table 4), plasma ANGPTL3 and ANGPTL4 levels were associated with age. The ANGPTL3 level had a positive correlation with age, while ANGPTL4 had a negative correlation. However, age was predictive for coronary atherosclerosis in the univariable analysis but not in the multivariable logistic regression analysis in the study. Research in a Finnish population sample also revealed a positive association of ANGPTL3 with age, in agreement with our results [32], while the association between ANGPTL4 and age in a previous study was contrary to that in ours [33]. These inconsistent results may be due to the small sample size and different constitutions of the sample population. A significant correlation between ANGPTL4 and age needs to be further studied in a larger sample.

This study also revealed the risks factors for atherosclerosis, such as age, sex, diabetes, smoking, and so on. It is well established that cigarette smoke exposure promotes vasomotor dysfunction directly, damages endothelial cells, induces tissue remodeling and prothrombotic processes and activates systemic inflammatory signals, which all contribute to atherogenic vessel wall changes [34]. ANGPTL3, ANGPTL4 and smoking status had a statistically significant association with atherosclerosis in both the univariate and multivariate analyses, but the levels of ANGPTL3 and ANGPTL4 were not different between smokers and nonsmokers. All these results suggested that ANGPTL3 and ANGPTL4 were better risk predictors of atherosclerosis independent of lipids, smoking, and other CHD risk factors that we studied.

### Study strengths and limitations

Previous research revealed both ANGPTL3 deficiency and ANGPTL4 abundance are associated with protection from CHD [35, 36]. Most study focus on variation and expression of ANGPTL3 or ANGPTL4 in CHD and AMI, less in atherosclerosis. This study comprehensively investigated the association between plasma ANGPTL3 or ANGPTL4 levels and coronary atherosclerosis severity, included the comparisons among different groups according to the degree of coronary stenosis and numbers of involved vessels. The results showed that ANGPTL3 was significantly increased and ANGPTL4 was decreased in the coronary atherosclerosis group. ANGPTL3 and ANGPTL4 levels were significantly associated with the severity of coronary vascular atherosclerosis. ANGPTL3 could play a possible role in the development of coronary atherosclerosis, and ANGPTL4 may be a protective factor against atherosclerotic plagues.

Some limitations of the present study should be recognized. First, it was conducted at a single center and the sample size was small. Our study participants were recruited from people with angina rather than general population. This design introduces possible bias into results and differences in baseline characteristics [37], so the findings may not be applicable to the general population. Second, the trial included participants who may have had a history of drugs or other diseases; hence, causality inferences cannot be made in the absence of data in the registries. Third, as numerous comparisons were performed with no adjustment for multiple testing, the increased risk of type I error should be acknowledged. Fourth, Heparin facilitates the release of LPL tethered to the endothelium into the blood stream and therefore there is more LPL present in post-heparin plasma [38]. The inhibitory effects of ANGPTL3 and ANGPTL4 on LPL in post-heparin plasma can be very different from those in pre-heparin plasma. Blood samples in this study were pre-heparin and the results may not be applicable in post-heparin plasma. Finally, considering the results of the study, they do not explain the causal relationships, only showing an association between ANGPTL3, ANGPTL4 and coronary atherosclerosis severity. It seems to be important to find the causal relationship arising from these findings, which requires future research.

## Conclusion

Increased ANGPTL3 and decreased ANGPTL4 exhibit an essential association with coronary atherosclerosis severity regardless of their lipid levels. ANGPTL3 may promote the development of atherosclerosis and ANGPTL4 may protect against atherosclerosis. They have a better prognostic value to predict coronary atherosclerosis risk with high sensitivity and specificity. ANGPTL3 or ANGPTL4 may become a convenient biomarker for screening and predicting coronary atherosclerosis in the future.

## Supplementary Information

**Additional file 1.**

## Data Availability

All data generated or analyzed during this study are included in this published article.

## Abbreviations

AMI: Acute myocardial infarction
ANGPTLs: Angiopoietin-like proteins
ANOVA: Analysis of Variance
apo: Apolipoprotein
ASO: Anti-sense oligonucleotide
AUC: Area under the ROC curve
BMI: Body mass index
BNP: Brain natriuretic peptide/ B-type natriuretic peptide
CHD: Coronary heart disease
CI: Confidence interval
DM: Diabetes mellitus
DOC: Distance on curve equaling square root of (1-Sen)^2^+ (1-Spe)^2^
EDTA: Ethylene Diamine Tetraacetic Acid
EL: Endothelial lipase
ELISA: Enzyme-linked immunosorbent assay
FBG: Fasting blood glucose
FFA: Free fatty acid
GHb: Glycosylated hemoglobin
HDL-C: High-density lipoprotein-cholesterol
HRP: Horseradish Peroxidase
HTG: Hypertriglyceridemia
IVUS: Intravenous ultrasound
LAD: Left anterior descending
LCX: Left circumflex
LDL-C: Low-density lipoprotein-cholesterol
LPL: Lipoprotein lipase
LVEDP: Left ventricular end diastolic pressure
LVEDP: Left ventricular end diastolic pressure
LVEF: Left ventricular ejection fraction
NYHA: New York Heart Association
OCT: Optical coherence tomography
ORs: Odds ratios
PBST: Phosphate Buffered Saline-Tween
RCA: Right coronary artery
ROC: Receiver operating characteristic curve
TTE: Transthoracic echocardiography
VLDL-C: Very low-density lipoprotein-cholesterol
