# Supplementary material for: Circulating ANGPTL3 and ANGPTL4 levels predict coronary artery atherosclerosis severity
Source: Lipids Health Dis. 2021 Nov 6;20:154. doi: 10.1186/s12944-021-01580-z (PMC8571829; doi:10.1186/s12944-021-01580-z)

**Supplementary Figure 1. the concentration (X) of standards against the mean absorbance (Y) of standards at 450 nm**


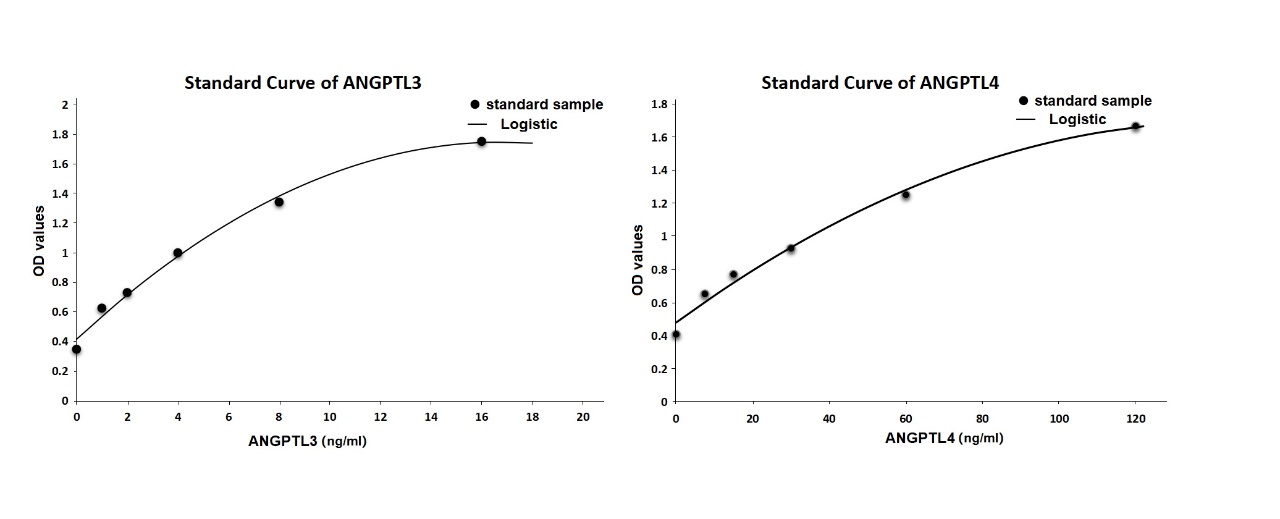


**Supplementary Table 1**. The level of ANGPTL3 or ANGPTL4 in the different coronary stenosis severity based on sex

| Coronary artery stenosis | ANGPTL3 (ng/mL) | | | ANGPTL4 (ng/mL) | | |
| --- | --- | --- | --- | --- | --- | --- |
|  | Male | Female | *P* value | Male | Female | *P* value |
| Degrees of stenosis |  |  |  |  |  |  |
| < 10% | 24.87±10.32 | 24.50±10.42 | 0.907 | 942.60±1258.42 | 826.36±673.12 | 0.807 |
| 10-50% | 55.79±93.90 | 48.62±36.63 | 0.499 | 458.52±287.53 | 471.15±254.34 | 0.582 |
| 50-75% | 55.02±36.85 | 45.88±19.63 | 0.620 | 466.92±338.79 | 482.01±259.29 | 0.258 |
| ≥ 75% | 47.87±35.84 | 62.31±43.58 | 0.265 | 407.13±235.96 | 404.79±117.23 | 0.394 |
| Numbers (stenosis ≥ 50%) |  |  |  |  |  |  |
| 0-vessel | 40.64±68.96 | 36.10±29.09 | 0.835 | 698.13±928.81 | 647.44±547.62 | 0.924 |
| 1-vessel | 57.93±41.18 | 56.48±33.75 | 0.617 | 432.54±327.45 | 493.44±207.02 | 0.010 |
| ≥ 2-vessel | 41.75±23.34 | 36.74±20.38 | 0.738 | 444.28±251.42 | 447.62±263.60 | 0.831 |

Values are expressed as mean ± standard deviation or percentage；ANGPTL: angiopoietin-like proteins;

**Supplementary Table 2**. Different factors affect ANGPTL3 or ANGPTL4 levels.

| Categorical Variables | Groups | ANGPTL3 (ng/mL) | *Z* value | *P* value | ANGPTL4 (ng/mL) | *Z* value | *P* value |
| --- | --- | --- | --- | --- | --- | --- | --- |
| Age, years | ≥ 65 | 44.95±28.57 | -3.637 | <0.001 | 541.10±717.23 | -2.410 | 0.016 |
|  | < 65 | 41.41±57.01 |  |  | 632.02±504.20 |  |  |
| Sex | Male | 45.89±56.31 | -0.641 | 0.522 | 576.66±719.42 | -1.447 | 0.148 |
|  | Female | 40.15±30.40 |  |  | 601.06±485.16 |  |  |
| Smokers | Yes | 48.10±66.13 | -0.242 | 0.809 | 555.81±347.93 | -0.740 | 0.459 |
|  | No | 40.41±28.96 |  |  | 606.16±720.89 |  |  |
| Alcohol consumers | Yes | 56.14±82.95 | -0.913 | 0.361 | 610.81±1009.19 | -0.829 | 0.407 |
|  | No | 39.58±27.34 |  |  | 582.45±458.13 |  |  |
| Hypertension | Yes | 46.30±53.49 | -1.797 | 0.072 | 541.78±453.35 | -2.032 | 0.042 |
|  | No | 38.25±29.54 |  |  | 659.54±799.80 |  |  |
| Diabetes mellitus | Yes | 44.03±33.92 | -0.860 | 0.390 | 473.39±306.35 | -1.583 | 0.113 |
|  | No | 42.85±48.42 |  |  | 620.28±674.16 |  |  |
| BMI >24kg/m^2^ | Yes | 44.88±53.47 | -0.918 | 0.359 | 656.40±668.22 | -0.499 | 0.617 |
|  | No | 39.85±31.53 |  |  | 614.77±537.42 |  |  |
| CHD | Yes | 46.07±29.53 | -1.830 | 0.067 | 504.99±294.57 | -0.275 | 0.783 |
|  | No | 42.26±49.31 |  |  | 612.46±679.49 |  |  |
| CHD family history | Yes | 43.07±54.73 | -2.044 | 0.041 | 562.25±450.79 | -0.142 | 0.887 |
|  | No | 43.32±28.31 |  |  | 626.10±797.75 |  |  |
| NYHA class III or IV | Yes | 41.93±24.83 | -0.347 | 0.728 | 385.04±184.40 | -1.946 | 0.052 |
|  | No | 43.21±47.01 |  |  | 605.87±636.75 |  |  |
| Atrial fibrillation | Yes | 49.84±37.82 | -1.253 | 0.210 | 588.35±452.53 | -0.478 | 0.633 |
|  | No | 42.48±46.30 |  |  | 588.51±629.79 |  |  |
| Hypolipidemic drugs | Yes | 47.99±79.78 | -0.126 | 0.900 | 613.00±566.72 | -0.329 | 0.742 |
|  | No | 41.88±32.16 |  |  | 582.46±634.69 |  |  |
| Aspirin | Yes | 43.83±47.02 | -1.205 | 0.228 | 587.42±606.97 | -0.239 | 0.811 |
|  | No | 34.26±21.04 |  |  | 601.67±734.15 |  |  |
| Hypoglycemic drugs | Yes | 43.86±35.51 | -0.536 | 0.592 | 481.68±321.43 | -1.240 | 0.215 |
|  | No | 42.94±47.63 |  |  | 612.00±661.75 |  |  |
| Antihypertensive drugs | Yes | 47.74±56.20 | -2.101 | 0.036 | 549.10±463.61 | -1.337 | 0.181 |
|  | No | 37.71±28.08 |  |  | 634.32±754.99 |  |  |
| HDL-C, mmol/L | ≤ 1.0 | 45.07±36.83 | -0.624 | 0.532 | 602.43±778.99 | -0.082 | 0.934 |
|  | > 1.0 | 42.07±53.95 |  |  | 575.46±482.87 |  |  |
| LDL-C, mmol/L | < 2.6 | 42.77±27.77 | -1.485 | 0.138 | 595.30±430.87 | -1.853 | 0.064 |
|  | ≥ 2.6 | 44.00±57.93 |  |  | 580.05±823.85 |  |  |
| Triglycerides, mmol/L | < 2.25 | 40.21±29.24 | -1.306 | 0.191 | 593.62±670.35 | -1.032 | 0.302 |
|  | ≥ 2.25 | 59.89±95.64 |  |  | 564.91±370.23 |  |  |
| FFA, mmol/L | < 0.6 | 46.97±53.96 | -0.532 | 0.595 | 594.98±758.43 | -1.127 | 0.260 |
|  | ≥ 0.6 | 50.93±43.58 |  |  | 465.78±344.38 |  |  |
| apoE, mg/dL | < 4.5 | 45.99±32.89 | -0.472 | 0.637 | 516.78±463.76 | -0.883 | 0.377 |
|  | ≥ 4.5 | 51.98±72.40 |  |  | 620.13±947.91 |  |  |
| Lipoprotein(a), g/L | < 0.3 | 45.61±49.47 | -3.625 | <0.001 | 565.59±629.30 | -1.351 | 0.177 |
|  | ≥ 0.3 | 31.28±29.85 |  |  | 711.76±649.11 |  |  |

Values are expressed as mean ± standard deviation or percentage; BMI: body mass index; CHD: coronary heart diseases; NYHA: New York Heart Association (2013 ACC/AHA guidelines); LDL-C: low-density lipoprotein-cholesterol; HDL-C: high-density lipoprotein-cholesterol; FFA: Free fatty acid; apo: apolipoprotein;

**Supplementary Table 3**. Correlation coefficient of ANGPTL3 and ANGPTL4 levels with other clinical characteristics and lipid parameters.

| Variable | ANGPTL3  Correlation coefficient | *P* value | ANGPTL4  Correlation coefficient | *P* value |
| --- | --- | --- | --- | --- |
| BMI | 0.028 | 0.635 | 0.010 | 0.857 |
| Age | 0.263 | <0.001 | -0.187 | 0.001 |
| GHb | 0.112 | 0.106 | -0.101 | 0.142 |
| triglycerides | 0.091 | 0.129 | 0.135 | 0.023 |
| HDL-C | -0.040 | 0.502 | 0.002 | 0.967 |
| LDL-C | -0.061 | 0.307 | 0.093 | 0.121 |
| Lipoprotein(a) | -0.178 | 0.003 | -0.003 | 0.958 |
| apoA-I | -0.023 | 0.739 | 0.060 | 0.391 |
| apoB | -0.095 | 0.170 | 0.093 | 0.180 |
| apoA-I/apoB | 0.008 | 0.904 | -0.020 | 0.775 |
| apoE | 0.080 | 0.252 | 0.115 | 0.101 |
| ANGPTL3 | - | - | -0.356 | <0.001 |
| ANGPTL4 | -0.356 | <0.001 | - | - |

BMI: body mass index; GHb: glycosylated hemoglobin; LDL-C: low-density lipoprotein-cholesterol; HDL-C: high-density lipoprotein-cholesterol; apo: apolipoprotein; ANGPTL: angiopoietin-like proteins;

**Supplementary Table 4**. Comparisons of diagnostic sensitivity and specificity of ANGPTL3 and ANGPTL4

| Biomarkers | ANGPTL3 | ANGPTL4 |
| --- | --- | --- |
| Cut-off value | 30.5 ng/mL | 497.5 ng/mL |
| AUC (95%CI) | 0.785 (0.736-0.836) | 0.334 (0.259-0.408) |
| DOC | 0.379 | 0.442 |
| Sensitivity (95%CI) | 0.712 (0.661-0.763) | 0.639 (0.565-0.713) |
| Specificity (95%CI) | 0.753 (0.702-0.804) | 0.745 (0.671-0.819) |
| *P* value | <0.001 | <0.001 |

ANGPTL: angiopoietin-like proteins; DOC, Distance on curve equaling square root of (1-Sen)^2^+ (1-Spe)^2^; AUC, Area under curve

**Supplementary Table 5**. Risk factors predicted by the cut-off value of ANGPTL3 or ANGPTL4.

| Categorical variables | ANGPTL3 (ng/mL) | | |  | ANGPTL4 (ng/mL) | | | | | |  |
| --- | --- | --- | --- | --- | --- | --- | --- | --- | --- | --- | --- |
|  | < 30.5 | ≥ 30.5 | *P* value |  | ≤ 497.5 | | | > 497.5 | | *P* value |  |
| Age, years |  |  | 0.002 |  |  | | |  | | 0.002 |  |
| ≥ 65 | 50 (38%) | 96 (56%) |  |  | 104 (55%) | | | 42 (36%) | |  |  |
| < 65 | 83 (62%) | 76 (44%) |  |  | 86 (45%) | | | 73 (64%) | |  |  |
| Sex |  |  | 0.915 |  |  | | |  | | 0.777 |  |
| Male | 68 (51%) | 89 (52%) |  |  | 99 (52%) | | | 58 (50%) | |  |  |
| Female | 65 (49%) | 83 (48%) |  |  | 91 (48%) | | | 57 (50%) | |  |  |
| Smokers |  |  | 0.934 |  |  | | |  | | 0.249 |  |
| Yes | 47 (35%) | 60 (35%) |  |  | 62 (33%) | | | 45 (39%) | |  |  |
| No | 86 (65%) | 112 (65%) |  |  | 128 (67%) | | | 70 (62%) | |  |  |
| Alcohol consumers |  |  | 0.705 |  |  | | |  | | 0.667 |  |
| Yes | 27 (20%) | 38 (22%) |  |  | 39 (20%) | | | 26 (23%) | |  |  |
| No | 106 (80%) | 134 (78%) |  |  | 151 (80%) | | | 89 (77%) | |  |  |
| Hypertension |  |  | 0.217 |  |  | | |  | | 0.043 |  |
| Yes | 75 (56%) | 109 (63%) |  |  | 123 (65%) | | | 61 (53%) | |  |  |
| No | 58 (44%) | 63 (37%) |  |  | 67 (35%) | | | 54 (47%) | |  |  |
| Diabetes mellitus |  |  | 0.618 |  |  | | |  | | 0.024 |  |
| Yes | 27 (20%) | 39 (23%) |  |  | 49 (26%) | | | 17 (15%) | |  |  |
| No | 106 (80%) | 133 (77%) |  |  | 141 (74%) | | | 98 (85%) | |  |  |
| Hypolipidemic drugs |  |  | 0.473 |  |  | | |  | | 0.733 |  |
| Yes | 29 (22%) | 32 (19%) |  |  | 37 (20%) | | | 24 (21%) | |  |  |
| No | 101 (78%) | 137 (81%) |  |  | 150 (80%) | | | 88 (79%) | |  |  |
| BMI ≥ 24kg/m^2^ |  |  | 0.612 |  |  | | |  | | 0.296 |  |
| Yes | 76 (58%) | 101 (61%) |  |  | 115 (62%) | | | 62 (55%) | |  |  |
| No | 56 (42%) | 66 (39%) |  |  | 72 (38%) | | | 50 (45%) | |  |  |
| CHD family history |  |  | 0.140 |  |  | | |  | | 0.976 |  |
| Yes | 84 (64%) | 95 (55%) |  |  | 112 (59%) | | | 67 (59%) | |  |  |
| No | 48 (36%) | 77 (45%) |  |  | 78 (41%) | | | 47 (39%) | |  |  |
| HDL-C |  |  | 0.917 |  | |  |  | | 0.894 | | |
| ≤ 1.0 mmol/L | 54 (45%) | 71 (44%) |  |  | | 80 (45%) | 57 (56%) | |  | | |
| > 1.0 mmol/L | 66 (55%) | 89 (56%) |  |  | | 98 (55%) | 45 (44%) | |  | | |
| LDL-C |  |  | 0.219 |  | |  |  | | 0.098 | | |
| < 2.6 mmol/L | 47 (39%) | 75 (47%) |  |  | | 84 (47%) | 39 (37%) | |  | | |
| ≥ 2.6 mmol/L | 72 (61%) | 85 (53%) |  |  | | 93 (53%) | 64 (63%) | |  | | |
| Triglycerides |  |  | 0.452 |  | |  |  | | 0.625 | | |
| < 2.25 mmol/L | 103 (86%) | 132 (83%) |  |  | | 150 (85%) | 85 (83%) | |  | | |
| ≥ 2.25 mmol/L | 17 (14%) | 28 (18%) |  |  | | 27 (15%) | 18 (17%) | |  | | |
| apoE |  |  | 0.311 |  | |  |  | | 0.554 | | |
| < 4.5 mg/dL | 47 (66%) | 79 (59%) |  |  | | 86 (60%) | 40 (65%) | |  | | |
| ≥ 4.5 mg/dL | 24 (44%) | 55 (41%) |  |  | | 57 (40%) | 22 (45%) | |  | | |
| Lipoprotein(a) |  |  | <0.001 |  | |  |  | | 0.189 | | |
| < 0.3 g/L | 90 (75%) | 144(91%) |  |  | | 152 (86%) | 82 (80%) | |  | | |
| ≥ 0.3 g/L | 30 (25%) | 14 (9%) |  |  | | 24 (14%) | 20 (20%) | |  | | |

BMI: body mass index; CHD: coronary heart diseases; LDL-C: low-density lipoprotein-cholesterol; HDL-C: high-density lipoprotein-cholesterol; apo: apolipoprotein; ANGPTL: angiopoietin-like proteins.

**Supplementary Table 6**. Different factors affected by diabetes mellitus or smoking status

| Categorical variables | Diabetes mellitus | | *P* value | Smoking status | | *P* value |
| --- | --- | --- | --- | --- | --- | --- |
|  | Yes | No |  | Yes | No |  |
| Age, years |  |  | 0.004 |  |  | 0.001 |
| ≥ 65 | 42 (64%) | 104 (43%) |  | 37 (35%) | 109 (55%) |  |
| < 65 | 24 (36%) | 135 (57%) |  | 70 (65%) | 89 (45%) |  |
| Sex |  |  | 0.051 |  |  | <0.001 |
| Male | 41 (62%) | 116 (48%) |  | 102 (95%) | 55 (28%) |  |
| Female | 25 (38%) | 123 (52%) |  | 5 (5%) | 143 (72%) |  |
| Alcohol consumers |  |  | 0.483 |  |  | <0.001 |
| Yes | 12 (18%) | 53 (22%) |  | 51 (48%) | 14 (7%) |  |
| No | 54 (82%) | 186 (78%) |  | 56 (52%) | 184 (92%) |  |
| Hypertension |  |  | <0.001 |  |  | 0.173 |
| Yes | 52 (79%) | 132 (55%) |  | 59 (55%) | 125 (63%) |  |
| No | 14 (21%) | 107 (45%) |  | 48 (45%) | 73 (37%) |  |
| Diabetes mellitus |  |  | - |  |  | 0.530 |
| Yes | - | - |  | 21 (20%) | 45 (23%) |  |
| No | - | - |  | 86 (80%) | 153 (77%) |  |
| Hypolipidemic drugs |  |  | 0.514 |  |  | 0.760 |
| Yes | 11 (18%) | 50 (21%) |  | 20 (19%) | 41 (21%) |  |
| No | 52 (82%) | 186 (79%) |  | 83 (81%) | 155 (79%) |  |
| BMI > 24kg/m^2^ |  |  | 0.063 |  |  | 0.165 |
| Yes | 45 (69%) | 132 (56%) |  | 69 (65%) | 108 (56%) |  |
| No | 20 (31%) | 102 (44%) |  | 38 (35%) | 84 (44%) |  |
| CHD family history |  |  | 0.748 |  |  | 0.329 |
| Yes | 40 (61%) | 139 (58%) |  | 67 (63%) | 112 (57%) |  |
| No | 26 (39%) | 99 (42%) |  | 40 (37%) | 85 (43%) |  |
| HDL-C |  |  | 0.123 |  |  | <0.001 |
| ≤ 1.0 mmol/L | 33 (53%) | 92 (42%) |  | 64 (66%) | 61 (33%) |  |
| > 1.0 mmol/L | 29 (47%) | 126 (58%) |  | 33 (34%) | 122 (67%) |  |
| LDL-C |  |  | 0.331 |  |  | 0.688 |
| < 2.6 mmol/L | 30 (49%) | 92 (42%) |  | 44 (45%) | 78 (43%) |  |
| ≥ 2.6 mmol/L | 31 (51%) | 126 (58%) |  | 53 (55%) | 104 (57%) |  |
| Triglycerides |  |  | 0.234 |  |  | 0.410 |
| < 2.25 mmol/L | 49 (79%) | 186 (85%) |  | 79 (81%) | 156 (85%) |  |
| ≥ 2.25 mmol/L | 13 (21%) | 31 (15%) |  | 18 (19%) | 27 (15%) |  |
| apoE |  |  | 0.348 |  |  | 0.048 |
| < 4.5 mg/dL | 31 (67%) | 95 (60%) |  | 47 (71%) | 79 (57%) |  |
| ≥ 4.5 mg/dL | 15 (33%) | 64(40%) |  | 19 (29%) | 60 (43%) |  |
| Lipoprotein(a) |  |  | 0.162 |  |  | 0.178 |
| < 0.3 g/L | 47 (78%) | 187 (86%) |  | 83 (88%) | 151(82%) |  |
| ≥ 0.3g/L | 13 (22%) | 31 (14%) |  | 11 (12%) | 33 (18%) |  |
| ANGPTL3 |  |  | 0.618 |  |  | 0.934 |
| < 30.5 ng/mL | 27 (41%) | 106 (44%) |  | 47 (44%) | 86 (43%) |  |
| ≥ 30.5 ng/mL | 39 (59%) | 133 (56%) |  | 60 (56%) | 112 (57%) |  |
| ANGPTL4 |  |  | 0.024 |  |  | 0.249 |
| < 497.5 ng/mL | 49 (74%) | 141 (59%) |  | 62 (58%) | 128 (65%) |  |
| > 497.5 ng/mL | 17 (26%) | 98 (41%) |  | 45 (42%) | 70 (35%) |  |

BMI: body mass index; CHD: coronary heart diseases; LDL-C: low-density lipoprotein-cholesterol; HDL-C: high-density lipoprotein-cholesterol; apo: apolipoprotein; ANGPTL: angiopoietin-like proteins.

**A certificate of language editing**


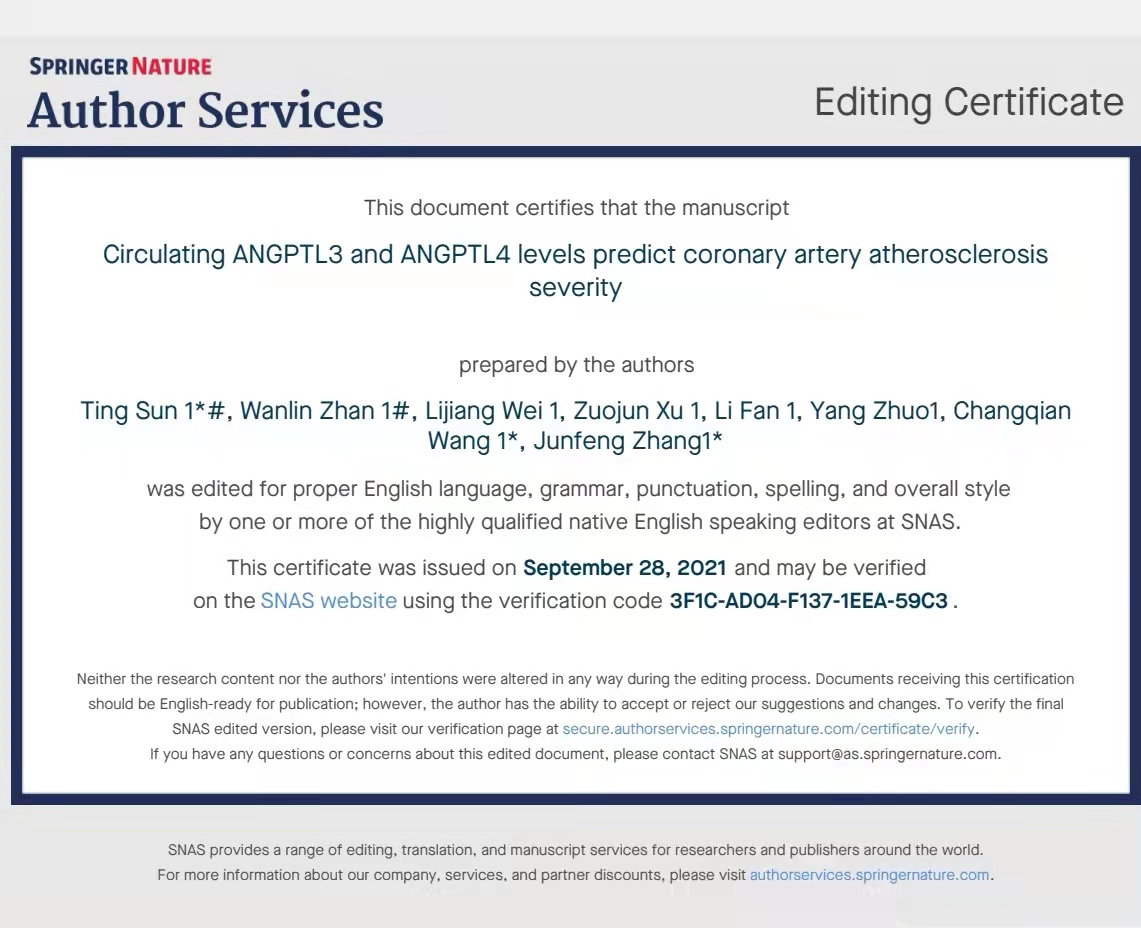


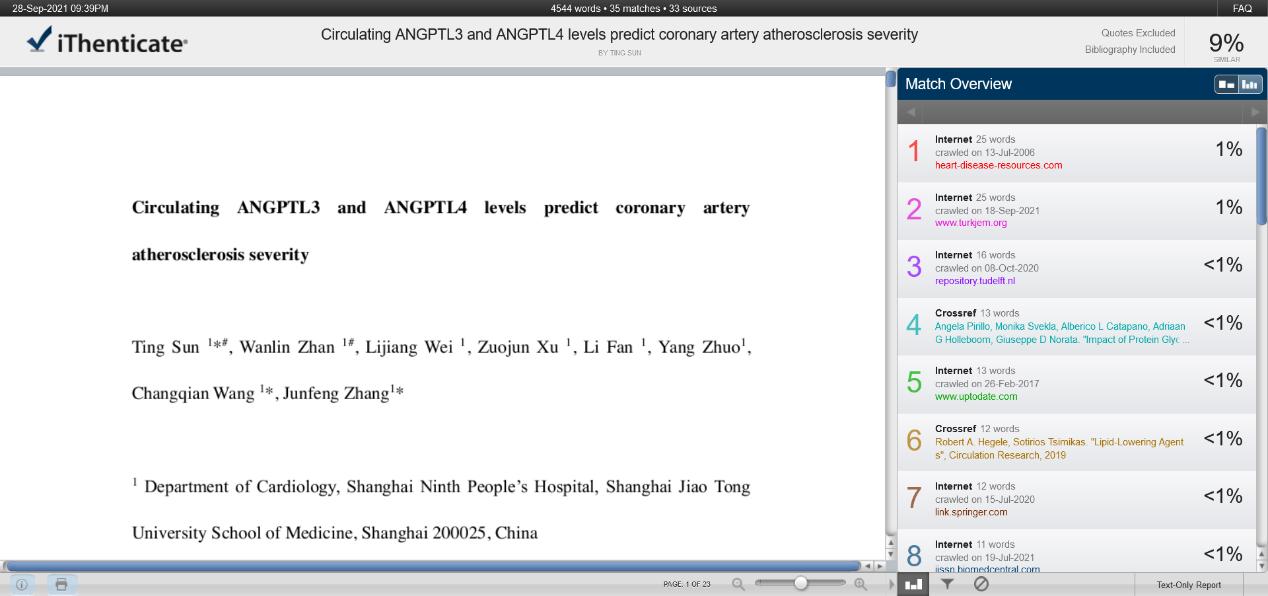


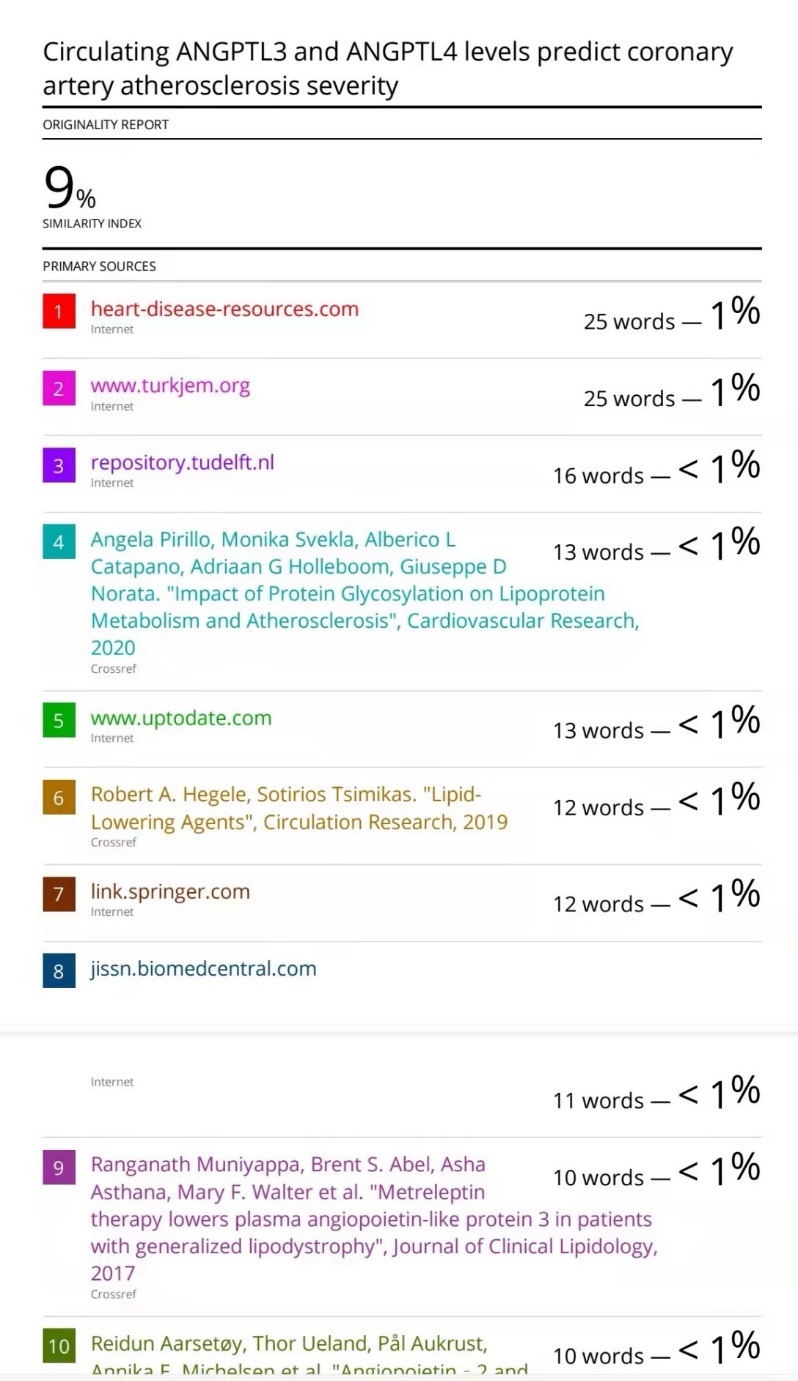

Supplement: Supplementary file 1 — Additional file 1. [file 12944_2021_1580_MOESM1_ESM.docx]
